# The Impact of Surface Roughness and Different Pre-treatments on the Shear Bond Strength of Super-translucent Multi-layered Zirconia to Adhesive Resin Cement: An In Vitro Study

**DOI:** 10.7759/cureus.78504

**Published:** 2025-02-04

**Authors:** Manar Almousli

**Affiliations:** 1 Department of Fixed Prosthodontics, Faculty of Dental Medicine, Damascus University, Damascus, SYR

**Keywords:** er: yag laser, multilayered zirconia, super-translucent zirconia, surface roughness, zircos-e solution

## Abstract

Purpose

The purpose of this in vitro study was to examine the impact of surface pre-treatment on the shear bond strength (SBS) of an adhesive resin cement to super-translucent multi-layered (STML) zirconia.

Materials and methods

Forty (n = 40) uniformly sized discs of STML zirconia (Katana, Kuraray, Noritake Dental Inc., Okayama, Japan), with dimensions of 3 mm in diameter and 3 mm in thickness, were produced using computer-aided design (CAD)/computer-aided manufacturing (CAM) technology and subsequently sintered following the manufacturer's instructions. The discs were subsequently divided into four equal groups (n = 10 each) based on the surface treatment method applied as follows: group 1 - control group with no treatment (Co); group 2 - sandblasting (SB); group 3 - Zircos Acids Zircos-E etching solution (BIO DEN CO, Seoul, Korea) (Z); group 4 - erbium-doped yttrium aluminum garnet (Er:YAG) laser. Surface roughness (Ra) was evaluated for each group. Round composite discs (Filtek Bulk Fill, 3M ESPE, St. Paul, MN), measuring 3 × 2 mm, were prepared for SBS testing and then bonded to the ceramic sample surface using composite resin cement (RelyX Ultimate Automix, 3M ESPE, Neuss, Germany). After securing the composite disc on the sample, each sample underwent an SBS test at a stress rate of 1 MPa/second with a force of 10 N. To ascertain the fracture type, fractured samples were inspected under a microscope. The ANOVA and post-hoc Tukey tests were employed to analyze differences in bond strength values among groups. All tests were conducted with a significance level of α = 0.05.

Results

Significant differences were observed in the SBS of cement applied to zirconium dioxide dental ceramics following various preparation methods. Statistically significant differences in the mean shear stress values (in megapascals) were found between the Z group and the Co group, as well as between the Er:YAG group and the other groups.

Conclusions

The application of Zircos-E for etching super ultra-translucent zirconia presents a promising approach to enhancing resin bonding, particularly in comparison to techniques involving Er:YAG laser and sandblasting.

## Introduction

Over the last decade, new technology development has moved in parallel with a fast evolution of metal-free ceramics on the rails of digital dentistry, opening new horizons in the field of prosthodontics [1].

Zirconia has proven itself to be an exceptional material in fixed prosthodontics, with high fracture toughness and impressive long-term survival in the oral environment [2,3]. Advancements in manufacturing methods, such as partial stabilization and the development of multi-layered zirconia, have paved the way for novel zirconia formulations with upgraded aesthetic qualities [4-6].

Among these, super-translucent multi-layered (STML) zirconia, characterized by a chemical composition of approximately 4.8 mol% yttria-stabilized tetragonal zirconia (Y-PSZ) with ~65 wt% cubic content, exhibits exceptional optical properties that closely mimic the translucency of natural teeth. This material demonstrates a graded translucency of up to 38% and possesses favorable mechanical properties, including a bending resistance of 748 MPa, alongside an average grain size of 2.81 (±0.17) μm [7-10]. This allows its use in three-unit anterior and posterior bridges or as single restorations, offering high aesthetics due to its light diffusion properties similar to natural teeth [11].

Although multilayered zirconia has high mechanical properties, its weak bonding to resin adhesives, due to its multi-crystalline nature, poses a significant challenge in dental applications, unlike lithium disilicate ceramics, which bond more effectively. Despite this, zirconia was chosen for this study because of its exceptional strength, fracture toughness, biocompatibility, and superior esthetics, making it ideal for high-stress dental restorations. While lithium disilicate ceramics bond more easily, they lack zirconia's durability for long-term use [12-14].

In silica-containing porcelain (gold protocol in adhesive), the inner surface is treated with water fluorine acid, and then the saline is used to achieve a permanent correlation between restoration and resin cement [15]. By contrast, the oxide porcelain (e.g., zirconium oxide) does not contain the silicate phase, and thus, the association with the dental surface cannot be achieved [16]. Therefore, it was necessary to have a standard and reliable adhesive protocol when using zirconia restorations, especially when dental preparation lacked the criteria for the persistence of restorations in the oral environment [17]; an effective adhesive protocol is not only critical for traditional zirconia restorations like crowns and bridges but also advantageous for bonded restorations such as laminate veneers, inlays, onlays, and overlays, these restorations rely heavily on strong adhesion to ensure their longevity and performance in the oral environment. By optimizing the bonding process, zirconia can be more effectively utilized in a variety of clinical applications, ensuring both functional durability and esthetic success [18,19].

Based on the results of numerous systematic studies that have proposed various surface treatment methods to enhance the adhesion between cement and zirconia ceramics [20,21], the aim is to achieve sufficient roughness to allow the resin cement to penetrate the resulting micro-retentions and achieve microscopic mechanical stability, in addition to the chemical bonding achieved by bonding agents (especially the use of 10-methacryloyloxydecyl dihydrogen phosphate (MDP)) [22,23]. Surface treatment methods can be divided into sandblasting, laser treatment, silica coating, silanization, or a combination of any of these methods [16]. Sandblasting remains the primary method for roughening the internal surface of zirconia restorations using aluminum oxide particles, and thus, it has been used as a reference for comparison with other surface treatment methods in various studies [24,25].

The erbium-doped yttrium aluminum garnet (Er:YAG) laser operates through a thermomechanical mechanism that focuses on vaporizing water within tissues. This vaporization process results in expansions and subsequent microscopic explosions, which effectively dislodge both organic and inorganic particles from the tissues. Consequently, this enhances the dentinal surface by revealing open tubules and eliminating the smear layer while also creating an irregular enamel surface. The resulting textured surface is essential for improving bonding effectiveness. Moreover, research indicates that this laser can be utilized to create irregular surfaces on various ceramic materials, which in turn enhances the bond strength between these ceramics and resin cements, further demonstrating its versatility and efficacy in dental applications [26].

In recent years, a notable advancement in dental materials has been the introduction of the Zircos-E etching agent, created by BIO DEN CO in Seoul, Korea, specifically for the chemical etching of zirconia. This liquid etching solution consists of a carefully formulated mixture that includes hydrofluoric acid (HF), hydrochloric acid (HCl), sulfuric acid (H₂SO₄), nitric acid (HNO₃), and phosphoric acid (H₃PO₄). When this solution is applied to zirconia, it effectively roughens the surface, thereby increasing the overall surface area through a preconditioning process. This modification enhances interfacial adhesion, which is crucial for improving the bond strength between zirconia and dental tissues. The development of the Zircos-E etching agent marks a significant step forward in restorative dentistry, enabling more reliable bonding methods for zirconia-based restorations [27,28].

The current study seeks to evaluate the impact of various surface treatments, including Zircos-E etching, on the resin bond strength with STML zirconia. The study is grounded in a null hypothesis that posits that the pre-treatment of the zirconia ceramic surface using Zircos and Er:YAG lasers will not influence the shear bond strength (SBS) of resin cement to the zirconia ceramic. Through this investigation, the research aims to provide insights into the effectiveness of these treatments in enhancing bond strength in dental applications.

## Materials and methods

Specimen preparation

A computer-aided design (CAD)/computer-aided manufacturing (CAM) milling system was utilized to fabricate forty disc-shaped samples (n = 40) from STML Katana zirconia (Kuraray Noritake Dental Inc., Okayama, Japan). The material composition of these samples is detailed in Table 1 [9]. The milled discs, in their pre-sintered state, were subjected to sintering in a high-temperature furnace (MIHM-VOGT GmbH & Co. KG, Studensee-Blankenloch, Germany) at 1550°C (2822°F). The sintering process involved a heating rate of approximately 10°C (18°F) per minute, followed by a two-hour holding period, as recommended by the manufacturer [9].

**Table 1:** Composition of Zircos-E, zirconia discs ZrO2: zirconium dioxide, Y2O3: yttrium oxide, HfO2: hafnium dioxide

Product	Composition	Manufacturer
Super-translucent multi-layered Katana zirconia CAD/CAM blanks	ZrO2 + HfO2: 88-93% yttrium oxide (Y2O3): 7-10%	Kuraray Noritake Dental, Kurashiki, Japan
Zircos-E etching solution	Hydrofluoric acid (HF) (0-25%), hydrochloric acid (HCl) (0-25%), sulfuric acid (H₂SO₄) (0-25%), nitric acid (HNO₃) (0-25%), phosphoric acid (H₃PO₄) (0-25%)	BIO DEN CO, Seoul, Korea

The dimensions of the discs were measured with a Sangabery 0-6 Electronic Digital Caliper (Sangabery, Shenzhen, China). Zirconia samples with cracks, chips, or irregularities on the bonding surface, as well as those deviating from the standardized dimensions by more than 3 ± 0.03 mm, were excluded. Each disc was rinsed in tap water for one minute and then soaked in a 90% ethanol solution. They were cleaned ultrasonically for 30 minutes using an ANGEL POS Ultra-Sonic Cleaner (model CD4820, 160W, Burnaby, Canada) and air-dried thoroughly afterward.

Based on the surface treatments applied, the cleaned discs were randomly split into four groups (n = 10). Group 1 consisted of control (as-sintered discs) (Co), which had no surface treatment, and group 2 consisted of sandblasted discs (SB), which were sandblasted with 110 μm Al2O3 (Cobra Aluoxyd, Renfert, Hilzingen, Germany) for 10 seconds at a pressure of 2.5 bar from a distance of 10 mm with the blasting nozzle moved gently perpendicular to the surface, then thoroughly rinsed with water. For group 3, the Zircos-E etching solution (BIO DEN CO, Seoul, Korea) (Z) was used to etch the discs for 30 minutes (as recommended by the manufacturer) in an ultrasonic cleaner (ANGEL POS Ultra-Sonic Cleaner, cd4860, 300W, Burnaby, Canada), the solution composition is listed in Table 1.

When handling the insertion and extraction of the specimens from the etching solution container, all necessary personal protective measures were rigorously observed, including the use of a mask, eyeglasses, and heavy-duty gloves. The etching solution container was accurately closed and placed in the ultrasonic cleaner, set to normal mode, at a temperature of 40ºC/104ºF. The discs remained undisturbed for a precise duration of 30 minutes. After this period, they were carefully removed and thoroughly rinsed under cold running water for two minutes. Following the rinsing process, the discs underwent steam cleaning and were then subjected to annealing at a temperature of 1150°C (2102°F). The annealing process included a holding time of 30 minutes at the specified temperature, as per the guidelines provided by the manufacturer. For group 4 (Er), to increase the laser energy absorption, the surfaces of the zirconia disks were coated with graphite powder (Fidelis Plus III, Fotona, Ljubljana, Slovenia) [12]. The laser was configured with specific parameters, including a wavelength of 2940 nm, a pulse duration of 50 µs (super short pulse (SSP)), an output power of 2 W, a pulse repetition rate of 10 Hz, and an energy density of 200 mJ. An R14 handpiece (Fotona, Ljubljana, Slovenia) equipped with an 800 µm sapphire tip was utilized, along with water:air spray ratio of 4:4. The sapphire tip was manually positioned at a distance of approximately 0.5 mm perpendicular to the surface of the disk. The entire surface of the zirconia disk was irradiated at a scanning speed of 3 mm/second for a duration of 10 seconds, operating in horizontal surface scan mode [29].

The forty discs, divided into four groups (Co, SB, Z, and L), were treated with 37% H₃PO₄ gel (Meta Etchant, Meta Biomed Co., Cheongju, Korea) for one minute to ensure thorough cleaning. Following this, the discs were rinsed with water and allowed to air dry. Subsequently, each group of discs was placed into separate containers filled with 90% alcohol and subjected to ultrasonic cleaning for 10 minutes. After the ultrasonic treatment, the discs were carefully removed from the containers and dried.

After finishing the surface treatment of zirconia discs, the samples were taken to the Faculty of Mechanical and Electrical Engineering at Damascus University to measure the surface roughness of the treated surfaces using a TR-200 handheld (the surface roughness tester). The measuring head was perpendicular to the surface of the cube. Then, three measurements were taken for each surface through mechanical contact between the measuring head and the sample surface, with each measurement being 3 mm long at a speed of 0.5 mm/second. The average of these three measurements was then taken to obtain Ra, representing each surface's average roughness.

Prior to conducting SBS testing, a liquid primer (G-Multi PRIMER, GC Corporation, Tokyo, Japan) containing MDP was applied to all samples using a microbrush to promote adhesion between the resin cement and the zirconia ceramics. A gentle airflow from an air syringe was applied for five seconds to facilitate the evaporation of the ethanol solvent.

Round composite disks measuring 3 × 2 mm (Filtek Bulk Fill, 3M ESPE, St. Paul, MN) were created using a mold. These disks were subsequently bonded to the sample surfaces using a composite resin cement (RelyX Ultimate Automix, 3M ESPE, Neuss, Germany), ensuring that the samples were properly prepared for subsequent testing and all the steps of cementations were done by the same operator to standardize the procedures as much as possible, following the bonding process, all the discs were securely positioned in acrylic bases to facilitate ease of handling during the application of the shear stress test.

Shear forces (SF) necessary for separating from the base were measured using the mechanical tester (Testometric, Rochdale, UK), whose head speed was 1 mm per minute. A knife edge was positioned at the adhesion interface to test the maximum SBS at the separation point between the zirconia and the resin cement. The metal housing containing the zirconia specimens was secured on the test base clamp using a 1000 N load cell, according to a previous study [30]. Specimens exhibiting gaps between the zirconia and the resin cement, or within the cement itself, were excluded from the study.

**Figure 1:**
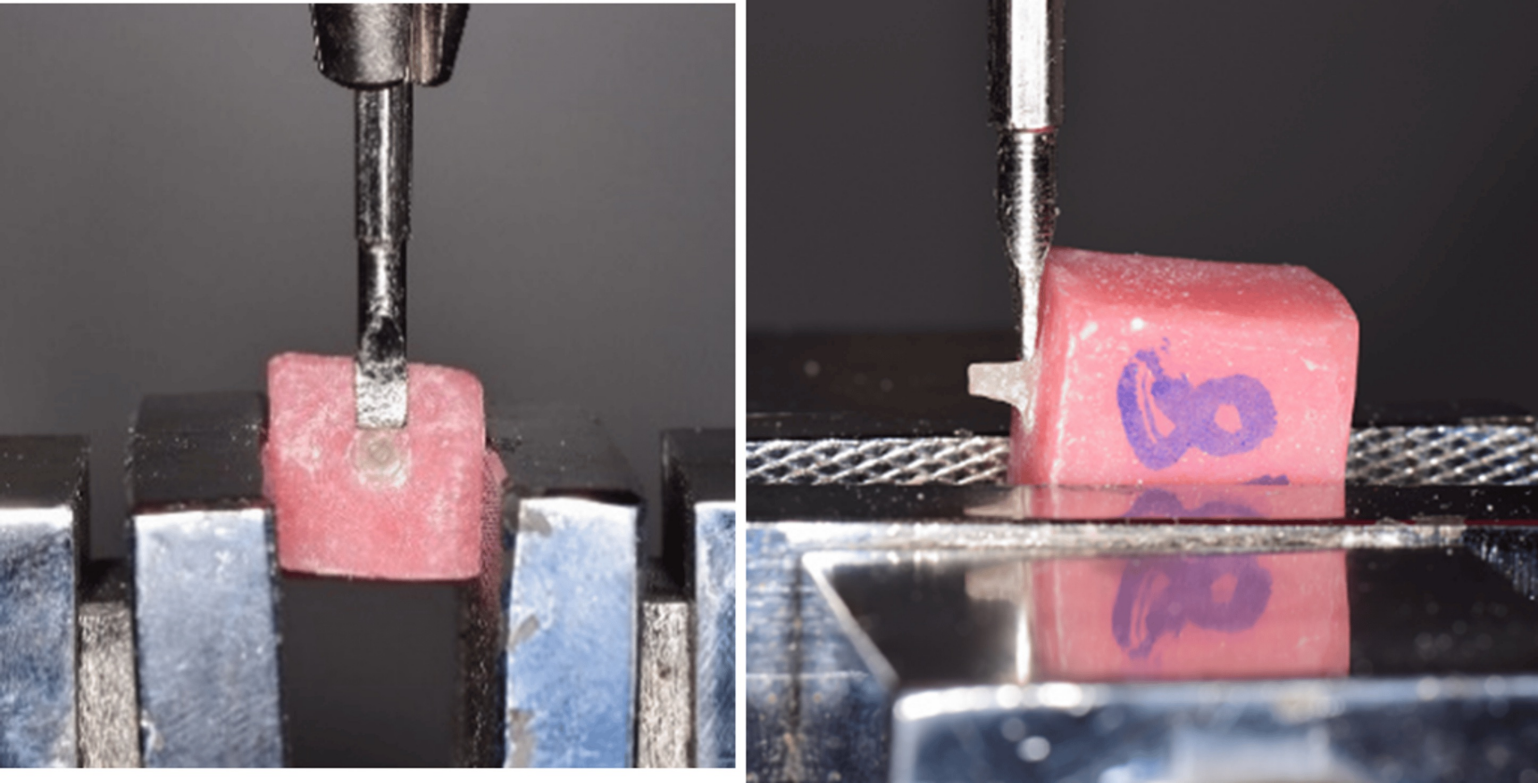
Positioning of the sample under shear stress

The applied forces (F) were measured in Newtons and subsequently divided by the surface area (SA) to calculate the shear stress (S) in megapascals (MPa) using the formula S = F/SA (Figure 1).

Following detachment, the outer surface of each zirconia sample, where the adhesive had been applied, was examined using an optical microscope at (30×). The purpose of this inspection was to assess the amount of residual adhesive using the adhesive remnant index (ARI), a scoring system designed to evaluate the integrity of the zirconia-cement interface. The ARI scale is defined as follows: a score of zero indicates that all the cement remains on the zirconia surface; a score of one signifies that more than half of the cement is retained on the surface; a score of two denotes that less than half of the cement remains on the surface; and a score of three indicates that no adhesive is left on the zirconia surface [31].

Statistical analyses

The statistical study involved conducting ANOVA to examine the differences between groups and a chi-square test to study the relationship between the failure mode and surface treatment. The statistical program is called SPSS (IBM SPSS Statistics for Windows, IBM Corp., Version 25, Armonk, NY).

Parametric tests were utilized after assessing normality with the Shapiro-Wilk test (p > 0.05).

## Results

Descriptive statistical study of zirconia disc roughness

The mean roughness values for each research group and other statistical variables were calculated and then organized in Table 2.

**Table 2:** Mean, standard deviation, minimum, and maximum values of zirconia disc surface roughness (in microns) Co: control; L: laser irradiation; SB: sandblasting; Z: acids

Studied Variable	Surface Treatment Method	Number of Discs	Mean	Standard Deviation	Minimum	Maximum	Shapiro-Wilk p
Roughness (in microns)	Co	10	0.07	0.02	0.03	0.11	0.514
	SB	10	0.15	0.09	0.02	0.28	0.648
	Z	10	0.19	0.13	0.01	0.48	0.392
	L	10	0.23	0.19	0.04	0.59	0.209

A one-way ANOVA analysis was performed to examine the impact of different surface treatment methods on surface roughness values across the research groups. It was observed that there was a lack of variance homogeneity among the groups as determined by the Levene test (p = 0.001). Consequently, the Welch test was utilized for further analysis. The results reveal a significance level below 0.05 (p = 0.007), indicating that, at a 95% confidence level, there are statistically significant differences in the mean surface roughness across the study groups. However, no pairwise comparisons were statistically significant, as denoted by the Games-Howell post-hoc analysis (p > 0.05). 

Shear bond strength

Mean SBS values (MPa), standard deviation, and minimum and maximum values for each group are listed in Table 3.

**Table 3:** The mean, standard deviation, minimum, and maximum values of shear stress measurements Co: control; L: laser irradiation; SB: sandblasting; Z: acids

Studied Variable	Surface Treatment Method	Number of Discs	Mean	Standard Deviation	Minimum	Maximum
Shear stress (in MPa)	Co	10	13.26	1.78	10.73	16.28
	SB	10	16.8	3.65	12.06	22.26
	Z	10	18.79	4.49	13.87	27.36
	L	10	8.71	3.5	3.37	13.76

One-way ANOVA (Table 4) showed statistically significant differences (p < 0.001) in mean SBS values between the four groups. Pairwise comparisons using Tukey post-hoc analysis (Tukey's honestly significant difference (HSD)) showed statistically significant differences in the mean shear stress values (in MPa) between the Co and the Z group (p = 0.006) and similarly between the L group and the Co group (p = 0.03) and between the L group and the SB and Z groups (p < 0.001). By analyzing the algebraic sign of the differences between the means, it is evident that the mean shear stress in the control (Co) group was lower than that in the Zircos-E (Z) group. Similarly, the mean shear stress in the laser (L) group was lower compared to the other groups.

**Table 4:** One-way ANOVA test for independent samples

Studied Variable		Sum of Squares	Degrees of Freedom	Calculated F Value	Significance Level	Significance of Differences
Shear stress (in MPa)	Between groups	586.67	3	15.99	<0.001	Significant differences
	Within groups	440.18	36			

ARI analyses

ARI scores presented in Table 5 showed that the Co and L groups showed a higher frequency of ARI = “3”. However, the sandblasting group showed a higher frequency of ARI = “2” scores, while the groups subjected to pre-treatment with Zircos-E showed an equal frequency of ARI scores = “0” and ARI = “1” scores.

**Table 5:** Frequencies (percentages) of the recorded failure mode in the research groups Co: control; L: laser irradiation; SB: sandblasting; Z: acids

Studied Variable	Surface Treatment Method	Number of Discs	0	1	2	3
Failure mode	Co	10	0 (0%)	2 (20%)	2 (20%)	6 (60%)
	SB	10	0 (0%)	4 (40%)	6 (60%)	0 (0%)
	Z	10	5 (50%)	5 (50%)	0 (0%)	0 (0%)
	L	10	0 (0%)	0 (0%)	4 (40%)	6 (60%)
	Total	40	5 (12.5%)	11 (27.5%)	12 (30%)	12 (30%)

Results of Fisher’s exact test showed that p < 0.001, suggesting that at a 95% confidence level, there is a relationship between the surface treatment method and the mode of failure.

## Discussion

Zirconia ceramics are highly regarded in the field of dental materials due to their excellent mechanical properties, biocompatibility, color stability, and low thermal conductivity, making them ideal for fixed prostheses, even in posterior regions. However, clinical failures, particularly debonding and loss of crown stability, remain significant concerns, with the latter being the second most common cause of failure in crowns and bridges [32].

The present study aimed to compare the effects of different surface treatments, including Zircos-E etching and Er:YAG laser irradiation, on the SBS of resin cement with STML zirconia. The null hypothesis, which proposed that pre-treatment of the zirconia ceramic surface using Zircos or Er:YAG lasers would not influence the SBS of resin cement to zirconia ceramic, was rejected in light of the study's results, the findings demonstrated that these pre-treatment methods did indeed have a significant impact on SBS.

Sandblasting or airborne particle abrasion (APA) is a crucial technique for enhancing the adhesion of zirconia restorations. By increasing surface roughness, removing contaminants, improving mechanical retention, activating the surface chemically, and ensuring consistent surface preparation, sandblasting significantly contributes to the successful clinical application of zirconia-based dental restorations [25,33,34].

APA, in conjunction with 10-MDP, has been demonstrated to significantly enhance the bond strength and durability of zirconia to resin cement [35].

This study further corroborates the positive impact of sandblasting on increasing zirconia-resin SBS, which agrees with several studies that investigated the influence of sandblasting on the bonding reliability of zirconia [20,36-38].

While our study has demonstrated that sandblasting enhances the interfacial integration of zirconia and resin, exploring alternative surface treatment methods could be both effective and warrant further development.

Our research has shown that the application of the Zircos-E etching system results in a mean SBS of (18.79 MPa) superior to other methods, such as air abrasion and Er:YAG laser irradiation, indicating that this treatment significantly enhances adhesion.

The SBS value of 18.79 MPa obtained in this study is notably close to the findings reported by Anh et al. [27], who recorded an SBS value of 19.85 ± 1.47 MPa for the zirconia surface architecture technique (ZSAT) group (Zircos-E). In their study, the ZSAT group demonstrated the highest SBS values among the tested groups, aligning with the results of the current investigation, which also identified the ZSAT group as achieving superior bond strength. This consistency across studies underscores the effectiveness of the ZSAT in enhancing the adhesive performance of zirconia restorations.

In the study by Cho et al. [30], the authors reported SBS values that are relevant to the findings of the current investigation. Specifically, they observed SBS values of 3.77 MPa, 7.42 MPa, and 16.15 MPa for groups treated with the Zircos-E etching system and bonded using Panavia F 2.0 (Kuraray Noritake Dental Inc., Okayama, Japan), RelyX Unicem (3M ESPE, Neuss, Germany), and Superbond C&B (Sun Medical Co., Ltd., Moriyama City, Japan), respectively. These results highlight the variability in bond strength depending on the adhesive system used, with Superbond C&B demonstrating the highest SBS among the tested materials. This aligns with the current study's emphasis on the importance of both surface preparation techniques and adhesive selection in achieving optimal bond strength for zirconia restorations.

Furthermore, the strong adhesion achieved through this pre-treatment highlights its clinical significance, particularly in ensuring the long-term durability of bonded restorations [30]. These findings align with previous studies, such as those conducted by Xie et al. [39], Lee Y et al. [40], and Lee MH et al. [41], which demonstrated that etching zirconia with higher concentrations of HF acid or a potent acid mixture of HNO₃ and HF significantly enhances SBS compared to conventional air abrasion techniques.

Also, we corroborate the findings of Ansari et al. [42], who demonstrated that the Zircos-E etching solution significantly enhanced the SBS of zirconia specimens.

However, these findings contrast with the results reported by Tsukakoshi et al. [43] and Zhao et al. [38], which found that air abrasion was a superior technique to enhance micro-mechanical retention; our results may be related to the preferential etching action of Zircos-E solution on the grain boundaries of super ultra-translucent zirconia. This etching process results in the formation of porosities with diverse shapes and depths, as the external atoms, which are more chemically reactive, are preferentially dissolved. This mechanism, which can lead to a reduction in grain size or even grain dislodgment, aligns with the description of zirconia etching provided by Sriamporn et al. [44].

Advancements in technology have driven the investigation of innovative systems capable of modifying the treatment and surface characteristics of ceramic materials, ultimately influencing their SBS with dental cement. Among these emerging alternatives, dental lasers have gained considerable attention and are being progressively incorporated into dental practices. Their potential to enhance surface properties and improve bonding efficacy has sparked substantial scientific interest, positioning them as a promising tool in modern dentistry [45].

Research suggests that when specific laser types are used with properly adjusted parameters, they can enhance surface roughness and modify surface characteristics, leading to improved bond strength in ceramics. The energy emitted by lasers can increase surface roughness by creating microcracks, which provide additional retention sites and enhance SBS [46]. However, in the case of zirconium dioxide (ZrO2) ceramics, laser treatment often does not achieve the desired level of surface roughness. The irregularities produced are typically shallow and lack the depth necessary for effective micromechanical retention, limiting their ability to improve bond strength. Additionally, debris generated during laser surface treatment can adhere to the molten ceramic surface, potentially reducing the quality of the bond [47].

Also, there is another downside. Excessive laser energy may compromise bond quality by melting the ceramic surface. Ural et al. demonstrated in their 2012 study that increasing the output power of a CO2 laser when treating zirconium oxide ceramics resulted in reduced SBS, and this decline was attributed to the creation of molten regions on the ceramic surface, which negatively impacted its bonding properties [46].

Previous studies have employed laser systems primarily designed for dental clinical applications, which often exhibit lower maximum output energy compared to the current system. Additionally, many studies necessitate the application of graphite powder to the zirconia surface, complicating the procedure and potentially dispersing laser energy. Furthermore, different laser beam types may exert varying influences on zirconia bonding properties due to differential laser energy absorption, such as in the case of CO2 lasers [48].

The current study demonstrated that Er:YAG laser energy adsorption induced surface modifications on zirconia, resulting in increased surface roughness, but this roughness on the surface of zirconia did not increase bond strength compared to conventional methods. The study by Komar et al. [45] supported these findings and further emphasized that while Er:YAG lasers can be utilized for surface treatment of alumina ceramics, the outcomes are significantly less effective compared to those achieved through etching. This contrasts with recent advancements in dental laser applications for zirconia surface treatment, where lasers have shown more promising results [49].

The findings of this study regarding the limited effectiveness of Er:YAG laser treatment are partially supported by recent research. A 2023 systematic review on zirconia bond strength durability noted that while laser treatments can be effective, their results can be variable and dependent on specific parameters [50]. 

This discrepancy could be attributed to differences in laser parameters, such as power settings, pulse duration, or wavelength. It highlights the need for further research to optimize laser treatment protocols for zirconia surface modification.

Both sandblasting and Er:YAG laser irradiation led to rougher surfaces. However, the laser-irradiated group (group L) exhibited a more pronounced increase compared to the sandblasted group (group SB). This discrepancy is attributed to the distinct mechanisms of energy transfer and absorption inherent to each technique. Nevertheless, the enhanced surface roughness did not correlate with a proportional increase in SBS.

The present study investigated only a single laser irradiation setting. Further research is necessary to identify the optimal laser parameters for maximizing resin-zirconia bond strength. Additionally, the impact of laser irradiation on the mechanical properties of zirconia, particularly fatigue resistance and aging behavior, warrants further investigation.

The second aim of this study was to analyze the ARI scores across four surface treatment methods: control, APA, Zircos-E etching, and Er:YAG irradiation. The ARI is a tool for evaluating the site of adhesive failure [51,52], and its use in this context offers important insights into the performance and failure patterns of each treatment method.

The control and laser-treated groups primarily exhibited adhesive failure at the interface between zirconia and resin cement, reflected by a higher ARI score of three, indicating weaker adhesive bonds. In contrast, specimens treated with Zircos-E acid showed a greater occurrence of ARI scores of zero and one, suggesting stronger adhesion. Meanwhile, the APA group displayed an ARI score of two, reflecting mixed failure modes with varying intensities. These findings align with the observations of Zandparsa et al. [53] and may be attributed to the enhanced adhesive performance of the resin cement, which proves advantageous for both sandblasted and etched super ultra-translucent zirconia surfaces.

However, a direct correlation between ARI and surface treatment was observed. This discrepancy highlights the complexity of adhesive bond strength, which is influenced by multiple factors beyond the mere location of failure. While ARI provides a useful qualitative assessment, it may not fully capture the quantitative aspects of bond strength.

Clinical relevance

The implications of these findings are significant for clinical practice. The enhanced bond strength achieved through effective surface pre-treatment methods can lead to improved longevity and performance of zirconia restorations. Clinicians should consider incorporating sandblasting or a combination of treatments to optimize bonding protocols, particularly in challenging cases where retention is critical. Furthermore, the study underscores the need for standardized adhesive protocols when using zirconia to ensure consistent results across different clinical scenarios.

Limitations and future directions

While the study provides valuable insights, it is essential to acknowledge certain limitations. The in vitro nature of the study may not fully replicate the complexities of the oral environment, including the effects of saliva, temperature fluctuations, and masticatory forces. Future research should aim to conduct long-term clinical trials to validate these findings in vivo. Additionally, exploring the synergistic effects of combining different surface treatments could yield further enhancements in bond strength.

Furthermore, the application of H₃PO₄ cleaning represents a potential limitation, as it may adversely affect zirconia bonding by modifying surface characteristics and weakening the adhesive interface. This emphasizes the necessity for future research to explore alternative cleaning techniques that preserve or enhance the bond strength of zirconia restorations without compromising their surface integrity.

## Conclusions

The findings demonstrate that surface treatment techniques for zirconia discs significantly influence both surface roughness and SBS, underscoring the necessity of choosing the right method to ensure optimal clinical performance. Among the evaluated methods, Zircos-E emerged as the most effective, improving SBS and offering valuable insights for clinical use. In contrast, pre-treatment with Er:YAG irradiation did not enhance bond strength when compared to traditional pre-treatment methods. By refining surface treatment protocols, dental practitioners can enhance the durability and reliability of zirconia restorations, leading to improved patient outcomes.
